# The Presence of CXCR4-Using HIV-1 Prior to Start of Antiretroviral Therapy Is an Independent Predictor of Delayed Viral Suppression

**DOI:** 10.1371/journal.pone.0076255

**Published:** 2013-10-01

**Authors:** Esther F. Gijsbers, Ard van Sighem, Agnes M. Harskamp, Matthijs R. A. Welkers, Frank de Wolf, Kees Brinkman, Jan M. Prins, Hanneke Schuitemaker, Angélique B. van ’t Wout, Neeltje A. Kootstra

**Affiliations:** 1 Department of Experimental Immunology, Sanquin Research, Landsteiner Laboratory and Center for Infectious Diseases and Immunity Amsterdam (CINIMA), Academic Medical Center, University of Amsterdam, Amsterdam, The Netherlands; 2 Stichting HIV Monitoring, Amsterdam, The Netherlands; 3 Department of Infectious Disease Epidemiology, School of Public Health, Imperial College London, London, United Kingdom; 4 Department of Internal Medicine, Onze Lieve Vrouwe Gasthuis, Amsterdam, The Netherlands; 5 Department of Internal Medicine, Center for Infectious Diseases and Immunity Amsterdam (CINIMA), Academic Medical Center, University of Amsterdam, Amsterdam, The Netherlands; Institut National de la Santé et de la Recherche Médicale, France

## Abstract

The emergence of CXCR4-using HIV variants (X4-HIV) is associated with accelerated disease progression in the absence of antiretroviral therapy. However, the effect of X4-HIV variants on the treatment response remains unclear. Here we determined whether the presence of X4-HIV variants influenced the time to undetectable viral load and CD4+ T cell reconstitution after initiation of cART in 732 patients. The presence of X4-HIV variants was determined by MT-2 assay prior to cART initiation and viral load and CD4+ T cell counts were analyzed every 3 to 6 months during a three year follow-up period. Kaplan-Meier and Cox proportional hazard analyses were performed to compare time to viral suppression and the absolute CD4+ T cell counts and increases in CD4+ T cell counts during follow-up were compared for patients with and without X4-HIV at start of cART. Patients harboring X4-HIV variants at baseline showed a delay in time to achieve viral suppression below the viral load detection limit. This delay in viral suppression was independently associated with high viral load and the presence of X4-HIV variants. Furthermore, the absolute CD4+ T cell counts were significantly lower in patients harboring X4-HIV variants at all time points during follow-up. However, no differences were observed in the increase in absolute CD4+ T cell numbers after treatment initiation, indicating that the reconstitution of CD4+ T cells is independent of the presence of X4-HIV variants. The emergence of X4-HIV has been associated with an accelerated CD4+ T cell decline during the natural course of infection and therefore, patients who develop X4-HIV variants may benefit from earlier treatment initiation in order to obtain faster reconstitution of the CD4+ T cell population to normal levels.

## Introduction

HIV-1 entry into target cells is mediated by binding of the viral envelope to CD4 and one of the coreceptors, CCR5 or CXCR4 [1]. The majority of HIV-1 infections is established by the outgrowth of viral variants that can only use CCR5 for entry [2-6]. CXCR4-using viral variants emerge during infection in at least half of patients infected with HIV-1 subtype B [7-10]. In the majority of patients, viral variants using CCR5 as coreceptor (R5) remain present in the viral quasispecies and coexist with viral variants using CXCR4 (X4) and variants capable of using both CCR5 and CXCR4 (R5X4) during the remaining course of infection [7,11].

In the absence of antiretroviral therapy, the emergence of R5X4 and X4 viral variants is associated with accelerated CD4+ T cell decline and more rapid disease progression [7-10,12-16]. While R5-HIV variants only infect activated memory CD4+ T cells, X4-HIV variants can additionally infect naïve CD4+ T cells that express CXCR4 but not CCR5 and therefore form a unique target cell population for CXCR4-using viral variants [7,17]. Loss of infected naïve CD4+ T cells will impair the generation of new activated effector and memory CD4+ T cells, which may explain the accelerated CD4+ T cell decline after the emergence of X4-HIV variants.

Whether the presence of X4-HIV before start of treatment influences the response to combination antiretroviral therapy (cART) is controversial [18-22]. Some reports describe a worse treatment response regarding viral load decline or CD4+ T cell increase in patients carrying X4-HIV variants [18-20], whereas other papers show similar rates of viral suppression for patients with and without X4-HIV variants upon start of antiretroviral therapy [21,22]. Here, we analyzed the effect of coreceptor switch before cART initiation on the response to antiretroviral therapy in a large cohort of 732 patients, of whom 31% carried X4-HIV variants at baseline, with a complete and reliable follow-up. The virological response to therapy and CD4+ T cell dynamics were analyzed during a three year follow-up.

## Methods

### cART treatment response

The response to cART in the presence or absence of X4-HIV variants was studied in 732 HIV-1 infected participants of the AIDS Therapy Evaluation in the Netherlands (ATHENA) cohort (n=726) who visited the Academic Medical Center or the Onze Lieve Vrouwe Gasthuis outpatient clinic in Amsterdam, or participated in the Amsterdam Cohort Studies (ACS) (n=6). Patients started first cART treatment between 1996 and 2009. For all patients, an MT-2 phenotypic assay was performed 12 to 0 months before start of cART. MT-2 assays were performed for study purposes (n=287) or at the request of the treating physician (n=445). Patients were followed for three years and viral load and CD4+ T cell counts were measured routinely every three to six months. The detection limit of HIV-1 RNA viral load in plasma was 1000 copies/ml for samples tested before 1997, 400 copies/ml in 1997 and 1998, and 50 copies/ml after 1998. Treatment failure was here defined as therapy switch due to virological failure, not achieving undetectable viral load within 3 years of follow-up, loss of follow-up or death before reaching an undetectable viral load.

### Ethics statement

The ATHENA observational cohort includes anonomyzed data from all HIV-infected patients living in the Netherlands who receive care in one of the 24 HIV treatment centers. Data were collected from all HIV-infected patients, who have been followed since 1996 in any of the centers. ATHENA patients are informed of data collection by their treating physician and patients can object to further collection of clinical data according to an opt-out procedure. Written informed consent and ethical approval is not obtained, as data collection is part of HIV care.

For our study, we also included data obtained from homosexual men (MSM) participating in the Amsterdam Cohort Studies (ACS). The ACS has been conducted in accordance with the ethical principles set out in the declaration of Helsinki, has been approved by the Medical Ethical Committee of the Academic Medical Center, and written informed consent was obtained from all participants prior to data collection.

### Natural history of X4-HIV emergence

To evaluate the emergence of X4-HIV variants during the natural course of HIV-1 infection, we studied 364 Caucasian homosexual men enrolled in the Amsterdam Cohort studies on the natural history of HIV-1 infection between October 1984 and March 1986 [23]. Of the 364 participants, 233 men were positive for HIV-1 antibodies at entry between October 1984 and April 1985. The remaining 131 seroconverted during the study. The participants with documented seroconversion and the seroprevalent participants with an imputed seroconversion date were analyzed as one study group, as previous studies have not revealed differences in AIDS-free survival between the two groups [24]. The development of X4-HIV was analyzed routinely at every visit using the MT-2 assay for 302 participants [25,26]. The censor date was set at the first day of effective antiretroviral therapy or end of follow-up of the participant.

### Analysis of coreceptor use

The MT-2 cell culture assay was used to determine the presence of X4-HIV variants in PBMC from HIV-1 infected patients during the natural course of infection or prior to start of cART as previously described [25,26]. In brief, MT-2 cells were cultured in Iscoves’ Modified Dulbecco’s Medium (IMDM) supplemented with 10% fetal calf serum (FCS), 100U/ml penicillin and 100µg/ml streptomycin. One million patient PBMC were co-cultivated with 1.6x10^6^ MT-2 cells in 5 ml medium for up to 4 weeks in duplicate and were monitored every 2-3 days for development of syncytia. The development of syncytia indicates the presence of CXCR4-using viral variants. Although, the MT2 assay cannot distinguish between R5X4 or X4 viral variants, we here define a positive MT-2 assay outcome as X4-HIV for simplicity.

### Data analysis

Baseline variables for patients with and without X4-HIV variants prior to start of cART are given as means and 95% confidence intervals (95%CI) or as numbers and percentages, and differences between the groups were determined using the Student’s T test, Fishers exact test or Chi square test (Table 1). Kaplan-Meier and Cox proportional hazard analyses were preformed to study the relation between the presence of X4-HIV variants prior to start of cART and time to reach undetectable viral load upon cART initiation. Univariate and multivariate relative hazards for time to achieve viral suppression were calculated for the presence of X4-HIV variants, viral load at baseline, CD4+ T cell count at baseline, the use of antiretroviral therapy (mono or dual therapy) prior to the initiation of cART and the calendar year when cART was initiated. Mean CD4+ T cell counts and the increase of the CD4+ T cell counts after start of cART were compared using the Mann-Whitney test.

**Table 1:** Baseline characteristics of patients with or without X4-HIV variants prior to start of cART.

	R5-HIV (n=508)	X4-HIV (n=224)	
	Number / Mean	Number / Mean	
	(Percentage / 95% CI^a^)	(Percentage / 95% CI^a^)	p value
**Age** (years)	39.77 (39.02-40.51)	40.14 (39.12-41.15)	0.58
**Gender** (Male)	440 (86.7%)	202 (90.2%)	0.22
**Nationality**			
Dutch	328 (64.6%)	139 (62.1%)	0.56
Other	173 (34.1%)	81 (36.2%)	0.61
Unknown	7 (1.4%)	4 (1.8%)	0.74
**Transmission route**			
Homosexual	316 (62.2%)	150 (67.0%)	0.24
Heterosexual	78 (15.4%)	32 (14.3%)	0.74
Intravenous	67 (13.2%)	24 (10.7%)	0.40
Other	31 (6.1%)	13 (5.8%)	1.00
Unknown	16 (3.1%)	5 (2.2%)	0.63
**HIV-1 subtype**			
B	210 (41.3%)	82 (36.6%)	0.25
Other	27 (5.3%)	9 (4.0%)	0.58
Unknown	271 (53.3%)	133 (59.4%)	0.15
**Therapy** (Experienced)^b^	149 (29.3%)	84 (37.5%)	**0.03**
**Treatment failure**			
Therapy switch due to virological failure	70 (13.8%)	34 (15.2%)	0.65
No undetectable viral load within 3 years	19 (3.7%)	8 (3.6%)	1.00
Loss of follow-up	5 (1.0%)	2 (0.9%)	1.00
Death	4 (0.8%)	2 (0.9%)	1.00
**Time since entry / seroconversion** (days)	1870 (1499-2241)	1559 (1345-1773)	0.29
**CD4+ T cell count** (/μl blood)	296 (279-313)	159 (139-179)	**<0.0001**
**HIV-1 viral load** (log_10_ copies/ml blood)	4.5 (4.4-4.6)	4.8 (4.7-5.0)	**<0.0001**

a 95%CI: 95% Confidence Interval.b Treatment experienced: use of mono or dual therapy before cART initiation.

We analyzed the development of X4-HIV variants during the course of infection and the absolute CD4+ T cell count at time of X4-HIV emergence in untreated participants of the ACS. The average yearly incidence of the development of X4-HIV variants during the natural course of infection was determined using the Kaplan-Meier survival analysis.

Statistical analyses were performed using Graphpad Prism version 5 and SPSS version 19.

## Results

### Baseline characteristics before start of cART

The response to cART in the presence or absence of X4-HIV variants was studied in patients who started cART between 1996 and 2009. Initiation of therapy occurred according to national guidelines in place at that time. MT-2 assays were performed to determine the presence of X4-HIV variants prior to start of therapy (median of 1.2 months prior, ranging from 0 to 12 months before start). Of the 732 HIV-positive patients under study, 508 (69%) had R5-HIV variants only, the remaining 224 (31%) harbored X4-HIV variants at the time of cART initiation.

Baseline characteristics of the patient population are shown in Table 1. The presence of X4-HIV variants at baseline was not associated with gender, age, nationality, route of infection, HIV-1 subtype or time since seroconversion or entry into the cohort at start of cART. Patients harboring X4-HIV variants had significantly lower CD4+ T cell counts at baseline (296 cells/µl versus 159 cells/µl, Table 1) and higher viral loads (4.5 log_10_ copies/ml versus 4.8 log_10_ copies/ml, Table 1). In addition, a significantly higher percentage of patients with X4-HIV variants at baseline had used mono or dual antiretroviral therapy prior to the start of cART (treatment experience; 29.3% versus 37.5%, Table 1).

The percentage of patients that switched therapy due to virological failure did not significantly differ for patients carrying R5-HIV and X4-HIV variants (13.8% versus 15.2%, Table 1). In total, 40 patients did not achieve treatment success: 27 patients did not achieve an undetectable viral load within three years of follow-up despite therapy switch; 7 patients were lost to follow-up before viral suppression below detection limits was documented; 6 patients died (3 AIDS-related deaths and 3 non-AIDS-related deaths) during the study. Treatment failure was not associated with the presence of X4-HIV variants at baseline (19.3% versus 20.5%, Table 1).

### The effect of HIV coreceptor use on viral suppression after initiation of cART

**Figure 1:**
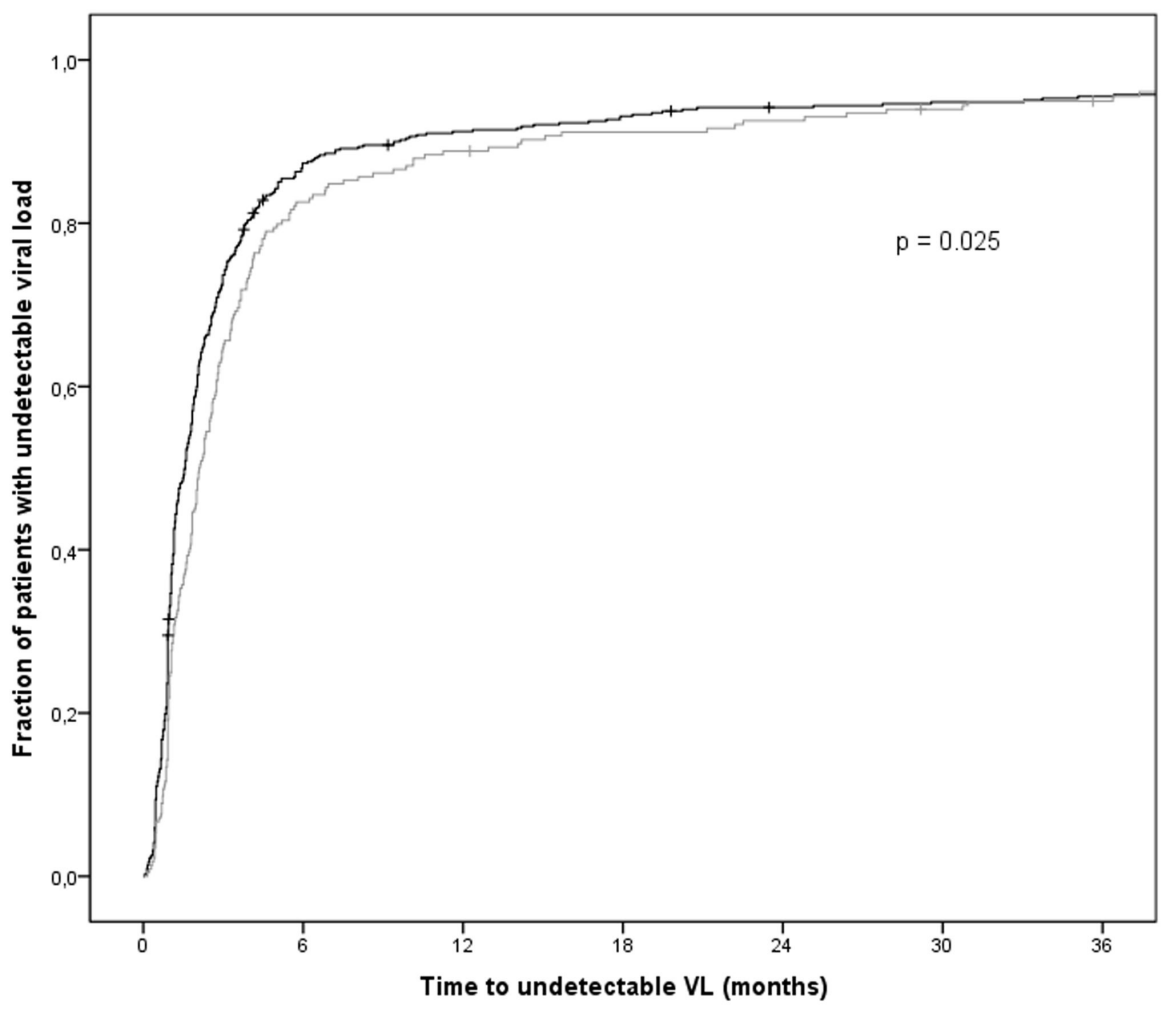
The presence of X4-HIV variants prior to cART is associated with a delay in time to viral suppression. Kaplan-Meier survival analysis for time to reach an undetectable viral load after initiation of cART. The black line represents patients with no detectable X4-HIV variants at baseline (n=508), the grey line represents patients with X4-HIV variants at baseline (n=224). Of the 732 patients in the analysis, 13 were censored (annotated by +) due to loss of follow-up (n=7) or death (n=6). At the end of the 3 year follow-up period, 27 patients had not yet reached viral load below the detection limit.

To analyze whether the presence of X4-HIV variants prior to start of cART influences the time to reach undetectable viral load, a Kaplan-Meier survival analysis was performed. Of the 732 patients, 13 patients were censored in the analysis (6 patients died and 7 patients were lost to follow-up) and 27 patients did not reach an undetectable viral load within the 3 years of follow-up (see Table 1). Patients harboring X4-HIV variants at baseline showed a delay in time to achieve viral suppression below the viral load detection limit (n=732, log-rank test p=0.025; Figure 1). For example, 90% of patients without X4-HIV reached undetectable viral load within 8.3 months after start of cART, while this took 14.1 months for patients with an X4-HIV variant. This indicates that the presence of X4-HIV at baseline is associated with a considerable delay in time to reach an undetectable viral load. However, a similar proportion of patients with R5-HIV and X4-HIV had achieved an undetectable viral load after 3 years of follow-up (94.5% and 94.6% respectively; Figure 1).

Univariate and multivariate relative hazard analyses were used to determine the association between baseline characteristics and time to achieve viral suppression. According to univariate analysis the presence of X4-HIV variants, baseline HIV-1 viral load above 4.5 log_10_ copies/ml [12], baseline CD4+ T cell counts below 200 cells/ml [12] and being treatment experienced were significantly associated with a delay in time to reach undetectable viral load (Table 2). Although during the study period the composition of cART regimens changed considerably, start of cART before or after the introduction of contemporary cART in 2002 did not influence the time to undetectable viral load (Table 2). In order to study the effect of all parameters combined, we performed a multivariate analysis. This analysis indicated that only the presence of X4-HIV variants at baseline and a baseline viral load above 4.5 log_10_ copies/ml were independently associated with a longer time to reach undetectable viral load (Table 2). Previous use of mono or dual therapy did not have an independent effect on the time to achieve viral suppression below the detection limit, suggesting that potential drug resistance did not influence the response to subsequent combination therapy.

**Table 2:** Uni- and multivariate analysis of baseline parameters on time to reach undetectable viral loads upon start of cART treatment.

	Univerate				Multivariate			
Baseline parameter	N^a^	Relative hazard	95% CI^b^	p value	N^a^	Relative hazard	95% CI^b^	p value
X4-HIV variants present	732	0.83	0.71-0.98	0.026	636	0.80	0.66-0.96	0.017
CD4 <200 cells/µl	701	0.74	0.64-0.86	0.0001	636	1.00	0.84-1.19	0.999
HIV-1 load >4.5 log_10_ copies/ml	636	0.45	0.38-0.53	<0.0001	636	0.45	0.38-0.54	<0.0001
cART initiation before the year 2002	732	0.91	0.76-1.09	0.31	636	1.04	0.86-1.27	0.670
Treatment experience	732	0.70	0.47-0.89	0.001	636	1.04	0.86-1.26	0.694

a N: number of patients.

b 95%CI: 95% confidence interval

### The effect of HIV coreceptor use on the reconstitution of CD4+ T cells after initiation of cART

**Figure 2:**
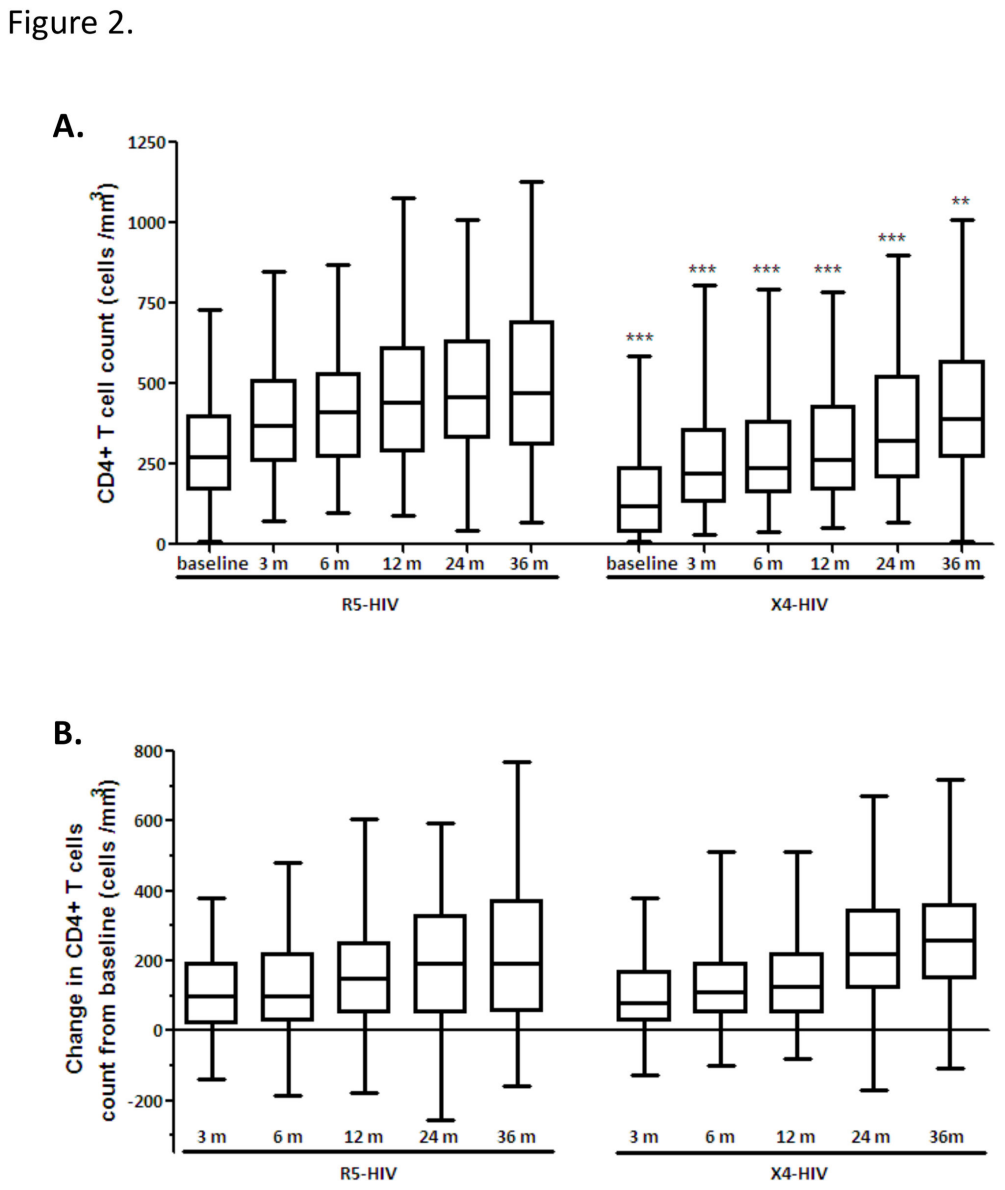
Increase in CD4+ T cell counts in response to cART treatment. A. Absolute CD4+ T cell counts at baseline (n=485 and n=216) and 3 (n=369 and n=180), 6 (n=372 and n=157), 12 (n=283 and n=128), 24 (n=247 and n=113) and 36 (n=241 and n=119) months after start of cART for patients with and without X4-HIV variants. Statistical significance was tested using the nonparametric Mann-Whitney test. Significance is indicated as follows: ** p<0.01 *** p<0.0001. B. Change in CD4+ T cell counts 3, 6, 12, 24 and 36 months after start of therapy compared to baseline CD4+ T cell count at start of treatment for patients with and without X4-HIV variants. Statistical significance was tested using the nonparametric Mann-Whitney test.

The effect of X4-HIV variants at baseline on the reconstitution of the CD4+ T cell pool was determined by analyzing the increase in CD4+ T cell counts after initiation of therapy. The absolute CD4+ T cell counts were significantly lower in the group of patients harboring baseline X4-HIV variants at all time points analyzed (Figure 2A). However, no differences were observed in the increases in absolute CD4+ T cell counts during the 3 year follow-up (Figure 2B), indicating that the reconstitution of CD4+ T cell counts in response to cART is independent of the presence of X4-HIV variants at baseline.

### HIV coreceptor switch in relation to the absolute CD4+ T cell count

**Figure 3:**
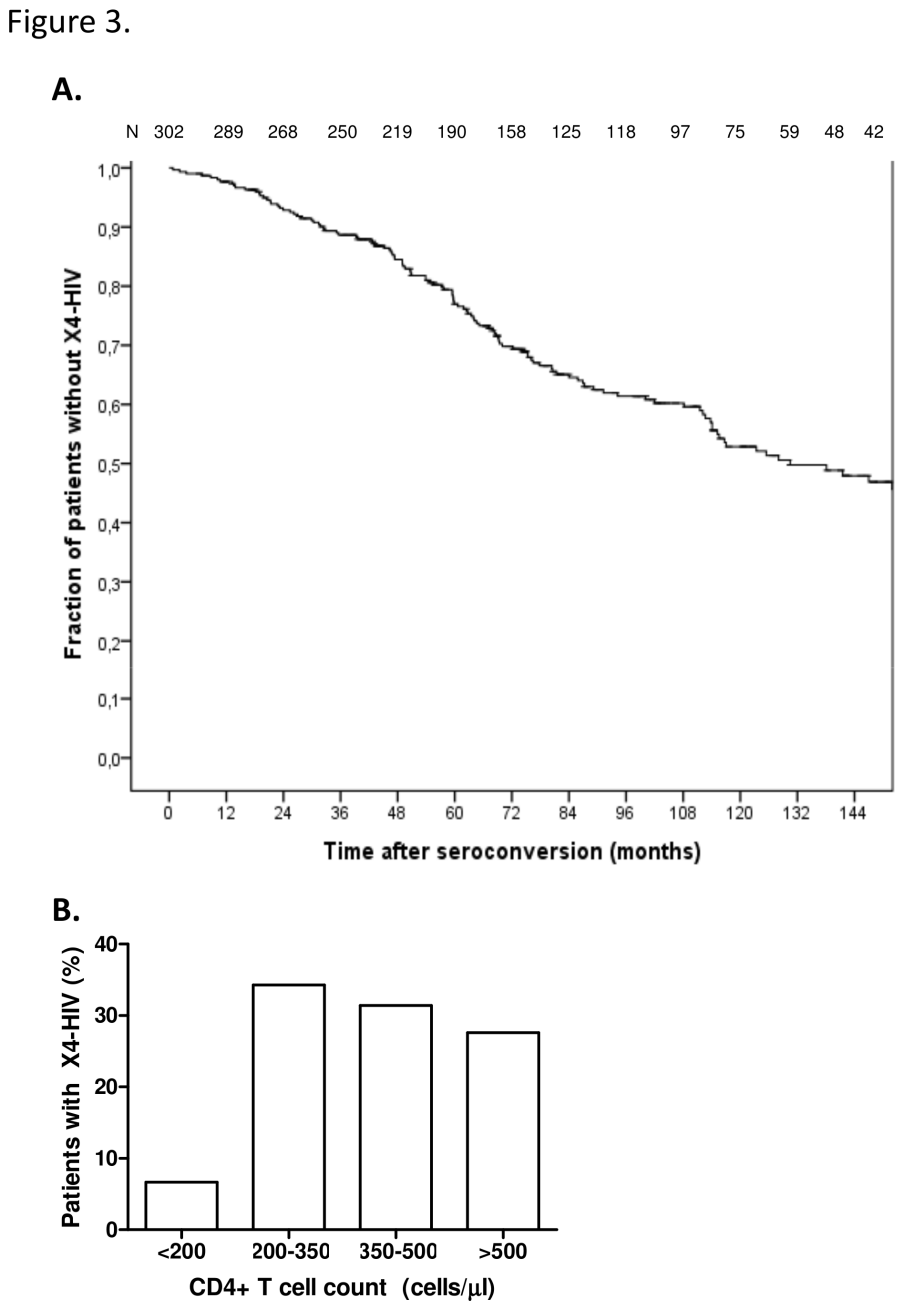
Emergence of X4-HIV variants in the ACS cohort. A. Cumulative fraction of patients harboring R5-HIV variants during HIV-1 infection. Numbers above the graph represent the number of patients in the analysis at that time. B. The percentage of patients that show X4-HIV emergence with absolute CD4+ T cell counts above below 200 cells/µl, between 200 and 350 cells/µl, between 350 and 500 cells/µl or above 500 cells/µl.

Current treatment guidelines in the Netherlands recommend initiating treatment at CD4+ T cell counts between 500 and 350 cells/µl. To determine whether this would sufficiently protect patients with X4-HIV variants from more severe immunological damage, we performed a detailed analysis of the emergence of X4-HIV variants during the natural course of HIV-1 infection in participants of the Amsterdam Cohort Studies (n=302). Although the moment of X4-HIV emergence after seroconversion differed largely between individuals, we observed a steady rate of X4-HIV development in time after seroconversion (Figure 3A), with an average incidence of 7.4% per year for a period of 12.5 years after seroconversion. When analyzing the CD4+ T cell counts at moment of X4-HIV emergence, we observed that in most cases X4-HIV emergence is observed when CD4+ T cell counts are between 200 and 350 cells/µl (34%). Interestingly, however, in 31% of patients X4-HIV variants emerged at CD4+ T cell counts between 350 and 500 cells/µl and in 28% of patients above 500 cells/µl. Therefore, in the majority of patients (59%), X4-HIV emergence occurs when CD4+ T cell counts are above 350 cells/µl (Figure 3B). Given that patients will experience a more pronounced loss of CD4+ T cells upon X4-HIV emergence, this suggests that their CD4+ T cell counts may drop well below 350-500 cells/µl before treatment is initiated.

## Discussion

We here studied the effect of the presence of X4-HIV variants at baseline on the cART treatment response. Patients harboring X4-HIV variants prior to start of cART show a delayed viral suppression compared to patients carrying R5-HIV variants only. In contrast, no significant differences were observed in the increase of CD4+ T cell counts upon start of cART during 3 years of follow-up or in the percentage of patients that experienced treatment failure. Our findings indicate that the rate of CD4+ T cell reconstitution is not influenced by the presence of X4-HIV variants at baseline.

The observed delay in viral suppression is independently associated with both high viral load and the presence of X4-HIV variants at baseline. Since X4-HIV can infect a wider range of target cells, such as naïve CD4+ T cells [17], these differences in cellular tropism of R5-HIV and X4-HIV variants may, in part, account for the delay in viral suppression associated with the presence of X4-HIV at baseline. Baseline CD4+ T cell count, treatment experience before start of cART and year of cART initiation were not independently associated with the time to achieve viral load suppression below the detection limit.

The increase in CD4+ T cell numbers in response to therapy during the three-year follow-up period was comparable between patients with or without X4-HIV variants, confirming previous reports [20-22]. However, a smaller CD4+ T cell increase during cART in patients with X4-HIV has also been reported [18,19]. In our study, patients harboring X4-HIV variants prior to initiation of therapy had significantly lower CD4+ T cell counts at baseline that remained significantly lower during the three years of follow-up. Even 5 years after start of cART the absolute CD4+ T cell counts in patients with X4-HIV variants had still not reached CD4+ T cell levels comparable to patients without X4-HIV variants (data not shown). However, one should note that not all patients included in this study had completed the 5 years of follow-up after start of therapy.

A similar finding was reported by Gras et al. who demonstrated similar increases in CD4+ T cell counts after start of therapy when stratifying patients for CD4+ T cell counts at baseline [27], also indicating that the absolute CD4+ T cell number at time of treatment initiation does not influence the rate of CD4+ T cell increase within the first years after cART initiation.

In the group of patients harboring X4-HIV variants, a relatively high proportion (37.5%) of patients was treatment experienced before start of cART. Previously, it was noted that patients carrying X4-HIV variants progressed to disease despite zidovudine monotherapy treatment, in contrast to patients with R5-HIV variants only, who showed no progression on zidovudine treatment [28]. However, the increased frequency of patients with X4-HIV variants in the group of treatment experienced individuals could not be explained by prior use of zidovudine monotherapy (data not shown). Although development of drug resistance due to previous use of monotherapy was not included in the analysis, treatment experience was not an independent predictor of time to viral suppression in the multivariate analysis. This suggests that drug resistance that may have been present did not influence the response to subsequent cART in our study.

Treatment guidelines and the composition of available antiretroviral medication are subject to change over time and most likely differed between patients that started cART in the late 1990’s and patients who started treatment after 2002 when contemporary cART was introduced. However, no independent association was observed for time to viral suppression and cART initiation before or after the year 2002.

Recently, treatment guidelines have changed and initiation of cART is recommended earlier in infection and at higher CD4+ T cell counts. However, X4-HIV emergence does not occur at one specific period in the course of HIV infection, but occurs with an average incidence of 7.4% per year. Moreover, in approximately 59% of patients X4-HIV emergence occurs at CD4+ T cell counts above 350 cells/µl and in approximately 27% of patients at CD4+ T cell counts above 500 cells/µl. This indicates that some patients will develop X4-HIV before start of therapy even under the current treatment guidelines.

In conclusion, the emergence of X4-HIV variants before initiation of cART is an independent predictor for delayed viral suppression after start of therapy. The risk of virological failure was comparable for patients with or without X4-HIV at baseline. Furthermore, CXCR4 coreceptor use of the virus does not influence the increase in CD4+ T cell counts upon start of therapy, confirming previous observations [20-22]. However, patients harboring X4-HIV variants do initiate cART with lower CD4+ T cell counts, which as a consequence results in lower CD4+ T cell counts in the first years after start of cART as compared to patients with only R5-HIV. Therefore, the assessment of HIV-1 coreceptor use has additive value in patient monitoring and care. Patients harboring X4-HIV variants could benefit from earlier initiation of cART and may need more frequent monitoring of CD4+ T cell counts to achieve faster suppression of viral replication and reconstitution of the CD4+ T cell population to normal levels.
